# Static magnetic field stimulation over motor cortex modulates resting functional connectivity in humans

**DOI:** 10.1038/s41598-022-11859-5

**Published:** 2022-05-12

**Authors:** Vanesa Soto-León, Mabel Torres-Llacsa, Laura Mordillo-Mateos, Carmen Carrasco-López, José A. Pineda-Pardo, Ana I. Velasco, Laura Abad-Toribio, Jesús Tornero, Guglielmo Foffani, Bryan A. Strange, Antonio Oliviero

**Affiliations:** 1 FENNSI Group, Hospital Nacional de Parapléjicos, SESCAM, Finca La Peraleda s/n, 45071 Toledo, Spain; 2 Universidad de Castilla la Mancha, Talavera de la Reina, Toledo, Spain; 3 IoTaP (Internet of Things and People), Malmö University, Malmö, Sweden; 4 HM CINAC (Centro Integral de Neurociencias Abarca Campal), Hospital Universitario HM Puerta del Sur, HM Hospitales, Madrid, Spain; 5 Universidad Alfonso X El Sabio, Villanueva de la Cañada, Madrid, Spain; 6 Hospital Los Madroños, Brunete, Madrid, Spain; 7 Neural Bioengineering Group, Hospital Nacional de Parapléjicos, SESCAM, Toledo, Spain; 8 Laboratory for Clinical Neuroscience, Centre of Biomedical Technology, Universidad Politécnica de Madrid, Madrid, Spain

**Keywords:** Neuroscience, Neuronal physiology

## Abstract

Focal application of transcranial static magnetic field stimulation (tSMS) over the human motor cortex induces local changes in cortical excitability. Whether tSMS can also induce distant network effects, and how these local and distant effects may vary over time, is currently unknown. In this study, we applied 10 min tSMS over the left motor cortex of healthy subjects using a real/sham parallel design. To measure tSMS effects at the sensori-motor network level, we used resting-state fMRI. Real tSMS, but not sham, reduced functional connectivity within the stimulated sensori-motor network. This effect of tSMS showed time-dependency, returning to sham levels after the first 5 min of fMRI scanning. With 10 min real tSMS over the motor cortex we did not observe effects in other functional networks examined (default mode and visual system networks). In conclusion, 10 min of tSMS over a location within the sensori-motor network reduces functional connectivity within the same functional network.

## Introduction

Static magnetic fields interfere with neural function in animals^1–7^ but currently, less is known about the effects of static magnetic fields on human brain function^8^.

Transcranial static magnetic field stimulation (tSMS) is a new non-invasive brain stimulation technique based on the transcranial application of a static magnetic field (120–200 mT at 2–3 cm from the magnet surface) over cortical areas. tSMS involves placing a compact high-powered neodymium (NdFeB) magnet on the scalp. Recently, we have performed a series of studies applying tSMS over different cortical areas, including visual, motor, supplementary motor and somatosensory cortex, and demonstrated a decreased excitability^9,10^ and a focal increase in the power of alpha oscillations in the underlying cortex^9,11–13^. Moreover, this neurophysiological effect of tSMS is functionally relevant, as it was paralleled by behavioural changes in humans^11,12,14^. Neurophysiological and behavioral tSMS effects have also been confirmed and extended by other research groups^10,15–28^.

Resting-state functional magnetic resonance imaging is a tool used to assess the organization and functional connectivity of brain networks. For this purpose, low-frequency (< 0.1 Hz) resting-state BOLD signal can be studied^29^. The temporal correlation of the resting state BOLD between different brain regions reflects functional connectivity; areas with a strong temporal correlation show high connectivity^30^.

Resting state networks (RSNs) reflect the brain's intrinsic functional connectivity^31,32^, and some of these networks are altered in different diseases^33–35^.

We have previously shown that tSMS of the supplementary motor area (SMA) increases local resting-state fMRI activity and bilateral functional connectivity between the SMA and both the paracentral lobule and the lateral frontotemporal cortex, including the inferior frontal gyrus^13^. It has been proposed that the functional connectivity of a network in the human brain is related to levels of inhibition in a major network node^36,37^. Since tSMS reduces motor cortex excitability^9,10,13,15,26^, we could thus expect a change of the functional connectivity of the Sensori-Motor Network (SMN) after tSMS application over the same region. The SMN was the first RSN to be identified by Biswal et al.^29^. The SMN includes cortical and subcortical areas, primarily the somatosensory cortex (postcentral gyrus) and motor (precentral gyrus) regions and extends to the supplementary motor areas.

More generally, it is not known whether tSMS over motor cortex induces neurophysiological changes distal to the application sites, and whether these are manifested as changes in long-range connectivity in other functional networks (e.g. default mode network). On the other hand, given previous observations of the time course of cortical excitability changes following tSMS^9^, we hypothesized that the functional connectivity would change over time. Specifically, we hypothesized that connectivity effects would be greatest immediately following the end of tSMS, and that this effect would diminish over time. The main goal of this study was, therefore, to evaluate the effects of motor cortex tSMS on the SMN.

## Materials and methods

### Subjects

We performed a total of 36 experimental sessions in 36 right-handed healthy subjects [11 males and 25 females, age (mean ± SD) 37.7 ± 12.3 years old; range 21–64]. Handedness was obtained from personal interview. The number of subjects to be included in each group was obtained from similar studies in the literature^9,10,15,26^. Data from two volunteers were excluded due to excessive motion artefact (absolute mean displacements greater than 0.2 mm). Thus, final analyses were performed on 34 subjects (10 males and 24 females, age 37.7 ± 12.5 years old; range 21–64). The participants were screened for history of hormonal, metabolic, circulatory, psychiatric and neurological disorders, and were medication-free at the time of the study. All participants gave their informed consent; the procedures had the approval of the institutional ethics committee (Toledo Area Ethical Committee for Clinical Research) and were conducted in accordance with the declaration of Helsinki.

### Experimental protocol

**Figure 1:**
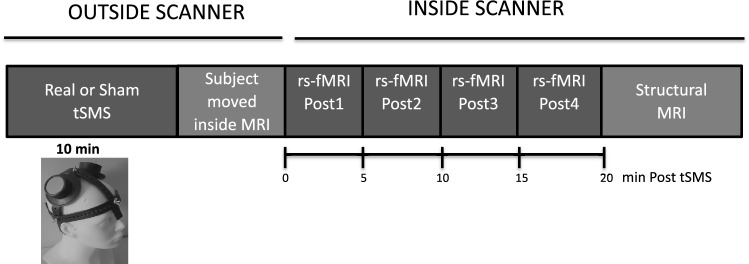
Experimental setup. All subjects underwent rs-fMRI after 10 min M1 real or sham tSMS. Rs-fMRI measurements were conducted in four blocks after tSMS application (out of scanner). The last rs-fMRI block (Post4) is considered a block without tSMS effect, the effects of 10 min tSMS were considered to be terminated by this time.

This protocol was designed to evaluate the effects in the functional human brain networks that are induced by tSMS. A schematic of the experimental protocol is shown in Fig. 1. This was a double-blind sham-controlled tSMS–rs-fMRI study with a parallel design. Subjects were tested under two conditions: real stimulation (n = 16; 5 males and 11 females, age 34.9 ± 10.8 years old; range 22–48) or sham stimulation (n = 18; 5 males and 13 females, age 40.2 ± 13.8 years old; range 21–64). There were no differences between the two groups with respect to sex (χ^2^, p = 0.824) or age (unpaired t test, p = 0.218). During the tSMS procedures, the participants were comfortably supine (on the same patient table platform used for MRI) in a semi-darkened location nearby, but outside, the MRI room. They were instructed to refrain from speaking, and to remain awake while in a calm, relaxed state. After the end of the stimulation period (10 min), the tSMS was removed and, subjects were moved into the scanner to test the effects of the tSMS on resting-state functional magnetic resonance imaging (rs-fMRI) activity. 20 min of rs-fMRI data were acquired in 4 sequential rs-fMRI blocks of 5 min each (Post1, Post2, Post3, Post4), continuously with no break in between. The subjects were instructed to be at rest, as motionless as possible, with their eyes open, and without engaging in any specific cognitive exercise during the entire scan. The electrophysiological after-effects produced by real tSMS tend to decay over time, concretely 6 min after the end of the stimulation^9^, and MRI data were collected for a sufficiently long time, such that the final MRI session (Post4) could be considered with reasonable confidence as representing a return to the state without stimulation effect. It is important to note that this protocol was designed to measure the effects of tSMS while eschewing any possible effect of exposing the subject to a 3 T MRI magnetic field *before* the tSMS^38^.

#### Transcranial static magnetic field stimulation (tSMS) of the motor cortex

For real tSMS, we used a cylindrical Nickel-plated (Ni-Cu-Ni) NdFeB magnet of 45 mm diameter and 30 mm of thickness, with a weight of 360 g (Model S-45-30-N; Supermagnete.de). The distance between the scalp and the motor cortex is about 15–20 mm^39^. At this distance, the magnetic field is about 120–200 mT^40^. For all subjects, the south magnetic field polarity was used. For the real group, the magnet was placed over the scalp position considered to be over the right-hand area of the left motor cortex (corresponding to the EEG c3 location, 10–20 international EEG system). We used a leather system to fix the magnet or sham cylinder (MAGlet45, Neurek SL, Toledo, Spain) over the motor cortex (Fig. 1). A steel metal cylinder was used for sham stimulation (MAG45s, Neurek SL, Toledo, Spain) over the left motor cortex in the sham group. This had the same size, weight and appearance of the magnet used for real tSMS. All subjects had a steel sham cylinder fixed over the contralateral (right) hemisphere (EEG c4 location) to counterbalance the weight. Subjects were not informed which side was stimulated and were asked if they thought it was the real intervention or the sham at the end of each session^9,10,12,14,41^; they were forced to choose “real” or “sham”. In previous studies we demonstrated that the subjects were not able to feel any sensation apart from the physical contact with the magnet.

#### Image acquisition

All subjects were scanned using a Siemens Magnetom Trio Tim 3 T scanner with a Siemens 12 channel Head Matrix Coil (Siemens Medical Solutions, Erlangen, Germany). Four rs-fMRI blocks were acquired during each session immediately after real or sham tSMS (Fig. 1), were acquired continuously without a break in between. Each of the four resting-state blocks was acquired using a gradient echo planar imaging (EPI) sequence with the following parameters: TR = 2500 ms, TE = 27 ms, FOV = 256 mm, flip angle = 90º, 32 slices, 64 × 64 matrix size, 4 mm slice thickness, 4 × 4 × 4 mm^3^ voxel size, 125 time points, 5:20 min duration. At the end of functional scanning, a T1-weighted sagittal MPRAGE structural scan was acquired with the following settings: TR = 2400 ms, TE = 2.98 ms, TI = 1000 ms, FOV = 238 × 200 mm^2^, flip angle = 8°, 176 slices, 238 × 200 matrix size, 1 mm slice thickness, 1 × 1 × 1 mm^3^ voxel size, 4:30 min duration.

#### Image analysis

Image pre-processing was carried out using FSL-FMRIB version 5.0.9^42^. T1-weighted images were processed to remove the non-brain tissue using BET^43^. The resulting brain-only image was segmented into three tissue types, grey matter (GM), white matter (WM) and cerebrospinal fluid (CSF) using FAST-FMRIB's Automated Segmentation Tool^44^. We created individual WM and CSF masks by thresholding the tissue probability maps at 0.7.

All volumes of rs-fMRI data were processed with the FEAT pipeline which involves slice timing and motion correction, brain extraction, high-pass temporal filtering (100 s) and linear registration to the standard MNI152 1 mm brain template. Registration from functional space to standard space was a two-step process, using a mid-point reference of a structural T1 image and concatenating the two steps to minimize resampling. First an average functional volume was registered to each subject’s T1 images using a global rescale transformation (7 degrees of freedom, DOF). Then the high-resolution T1 images were registered to the MNI152 template using an affine transformation (12 DOF). Finally, both transformations were concatenated and applied to the rs-fMRI data, which finally was smoothed using a Gaussian kernel (6 mm FWHM).

Variance that could be explained by known confounds was removed from each voxel of the rs-fMRI time series. Nuisance regressors included the mean-centred global WM and CSF signal intensities, the six head motion parameters, their squared values as well as their first-order derivatives^45^.

#### Resting state functional connectivity analysis

A seed-based connectivity analysis (SCA) was used to examine the functional connectivity of rs-fMRI data^46–48^, carried out using FSL-FMRIB version 5.0.9^42^.

We defined three seeds in three regions of interests (ROIs) each pertaining to the Sensori-Motor Network (SMN), Default Mode Network (DMN)^49^ and Visual Network (VN), respectively. The visual network (VN) identified by rs-fMRI includes both striate cortex (V1, Brodmann area 17) and many extra-striate areas in the occipital lobe. The VN occupies a large fraction of the posterior cortical surface. The default mode network (DMN) involves the posterior cingulate cortex, medial prefrontal cortex, and lateral parietal cortex. Activity in the default mode network increases when the individual is in the resting condition. It is also known as the task-negative network because it becomes less active when the individual engages in some tasks. This network is also known as the mentalising network due to its participation in social cognition such as introspection, mind wandering, emotional processing, thinking others mental state^50–53^. The SMN includes many cortical and subcortical areas, primarily the somatosensory cortex (postcentral gyrus) and motor (precentral gyrus) regions and extends to the supplementary motor areas, premotor cortex and cingulate motor areas, second somatosensory area, granular insula, posterior parietal cortex, cerebellum, basal ganglia and thalamus.

We therefore tested for tSMS-induced effects within the tSMS stimulated network (SMN) and in two networks that are not primarily related to the stimulated area (namely, DMN and VN).

**Figure 2:**
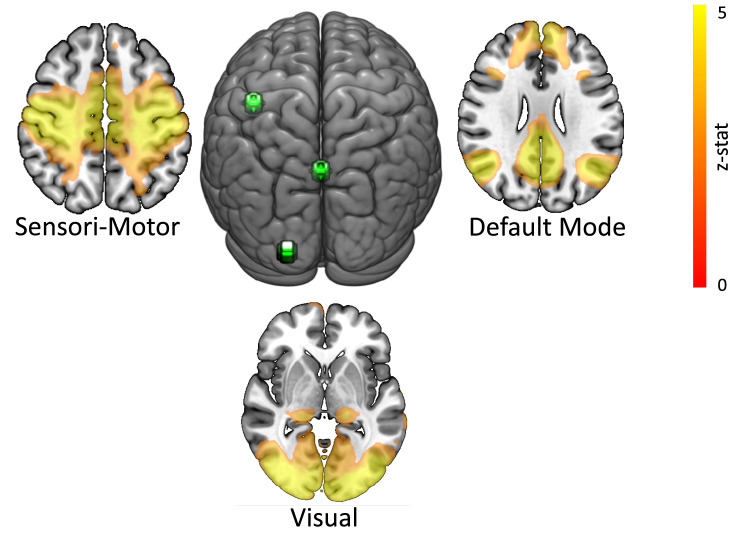
Functional connectivity and resting-state networks at 15–20 min after sham tSMS stimulation, block without tSMS effect. The three functional connectivity networks [Sensori-Motor Network (SMN), Default Mode Network (DMN) and Visual Network (VN)] are displayed along with the corresponding seed locations (left-M1: − 40, − 18, 48, PCC: 0, − 54, 26 and left-V1: − 20, − 100, 4) that were used to define the networks with the seed-based connectivity analysis (SCA).

ROI seeds consisted of a sphere with a 5 mm radius. For the SMN network, which includes the primary motor cortex (M1), the anatomically defined seed was centered on the “hand knob” region of the Rolandic sulcus of the left hemisphere (left-M1: − 40, − 18, 48)^54,55^. For the DMN, a single seed was placed in posterior cingulate cortex (PCC: 0, − 54, 26) based on coordinates provided from other rs-fMRI studies^51^. For the VN, we used the primary visual cortex (left-V1: − 20, − 100, 4) as seed region, with the seed coordinate defined from group activation maxima based on a visually cued sensorimotor task described by^56^ (Fig. 2).

The mean time series of the seed-region voxels was used as a regressor in 3 general linear models (GLM), in order to calculate whole-brain, voxel-wise functional connectivity maps (fcMap) of covariance with the 3 different seed regions (left-M1, PCC, and left-V1, respectively). Thus, these whole-brain analyses allowed us to extract the SMN, VN and DMN, identifying regions whose BOLD signal is temporally correlated with the seeds used^49^ (Fig. 2).

Connectivity maps were obtained for each individual and for each rs-fRMI acquisition (4 acquisitions: Post1, Post2, Post3, Post4). This step was repeated with the three seeds defined (left-M1, PCC, left-V1). Therefore, all subjects had 12 fcMaps. The resulting z statistic images depicting functional connectivity were thresholded at z > 2.3 and a corrected cluster extent threshold of p = 0.050 applied^57^.

Given previous observations of the time course of the cortical excitability changes following tSMS^9^, we hypothesized that SMN connectivity would change over time. We modelled the effect of tSMS over time as a linear decrease, that is, the effect decreases over time (Post1 > Post2 > Post3 > Post4). Our primary comparison of interest was a group (Real, Sham) by time (linear change) interaction. This comparison was performed separately for connectivity maps resulting from using seed regions left-M1, PCC and left-V1. Results are considered significant if they meet a cluster threshold of z > 2.3, p = 0.050 corrected for multiple comparisons^57^.

The ensuing maxima of the group by time interaction were identified and the mean measure of functional connectivity strength for a sphere (5 mm-radius) centered at this location was then extracted for each subject for each session (Post1, Post2, Post3 and Post4) and subject (real or sham tSMS), separately. Functional connectivity was evaluated using a repeated-measures ANOVA with Time (Post1, Post2, Post3 and Post4) as within-subject variable and Stimulation (real, sham) as between-subject factor. In case of a significant interaction, follow-up ANOVAs for each Stimulation condition, and post hoc tests (Bonferroni corrected) were performed. Data were analyzed using IBM SPSS Statistics version 26.0.

#### Resting state frequency domain analysis

We analysed the spectral properties of the ROI (seed) pertaining to the SMN (where the tSMS was applied). The frequency distribution of time courses for the ROI was evaluated by computing the power spectral density (PSD) of each subject's ROI time course. The power spectrum of the time course was generated using Welch’s method in Matlab version R2014b, which estimates the PSD of the input signal dividing it into eight sections of equal length, each with 50% overlap. The rs-fMRI run was composed of 125 time points (functional volumes), then the length N of the FFT = 64 points, with overlap window of 32 points. This setting produced a high resolution of specific observation of the power spectrum density with 33 bins of 0.00625 Hz, ranging from 0 to 0.2 Hz. The individual peak frequency (IPF) was estimated from the PSD of each subject and the area below the curve within the frequency range of the IPF ± 0.0125 Hz was individually calculated.

Given previous observations of the time course of the cortical excitability changes following tSMS^9^ described above, we hypothesized that the seed region PSD would change over time. Again, we modelled the effect of tSMS over time as a negative linear effect, that is, the effect decreases over time (Post1 > Post2 > Post3 > Post4). Our comparison of interest was a group (real, sham) by time (linear changes) interaction, evaluated using a repeated-measures ANOVA with Time (Post1, Post2, Post3 and Post4) as within-subject variable and Stimulation (real, sham) as between-subject factor, followed by follow-up ANOVAs for each Stimulation condition in case of significant interaction and post hoc test (Bonferroni corrected). This comparison was only performed for PSD calculated from the left-M1 seed region (stimulated region). Data were analyzed using IBM SPSS Statistics version 26.0.

## Results

Thirty-four healthy subjects received real (n = 16) or sham (n = 18) tSMS. The experimental procedure was well tolerated. None of the subjects needed to interrupt or terminate the session due to side effects. Subjects were blind to stimulation type received (real, sham) and forced choice questioning after MRI scanning did not show significantly correct identification of the real magnet *vs* sham sessions (χ^2^ = 3.031, p = 0.081).

### Resting state connectivity domain analysis

**Figure 3:**
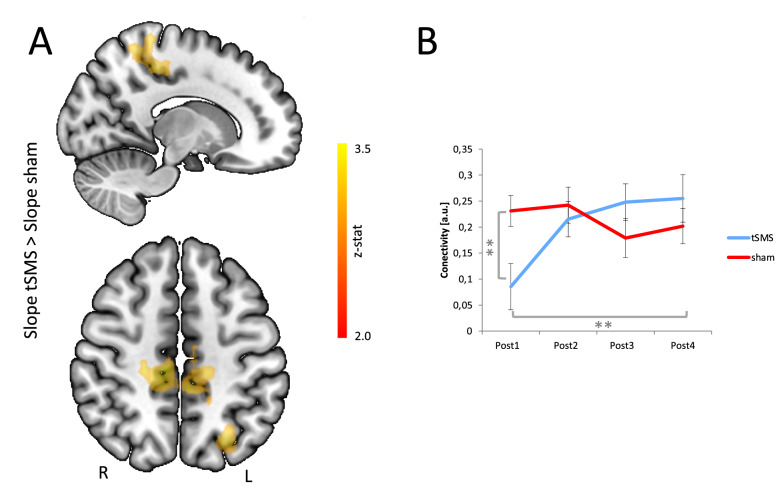
Stimulation by time resting state connectivity analysis. (**A**) Anatomical maps of the comparison between linear slope contrast in the real group and slope contrast in the sham group. Maps determined by z > 2.3 and a (corrected) cluster significance threshold of p = 0.05. (**B**) Time course of the connectivity between the left-M1 seed and the area in which the greatest significant difference occurred when comparing the slope contrast between the two groups (Broadman Area 7). In addition, there is an increase in connectivity over time in the real group. Error bars depict standard error of the mean (SEM). **p < 0.010. *a.u.* arbitrary unit.

Our primary comparison of interest was a time dependent change in functional connectivity following application of tSMS relative to sham stimulation. That is, we performed a seed-based whole-brain group analysis, entering linear change over sessions Post1 to Post4 as a regressor. Taking left-M1 (stimulated region) as a seed region, we observed a significant group by time interaction (cluster z > 2.3, p < 0.050) in a bihemispheric SMN (Fig. 3A). This cluster was within the SMN, and extended into the DMN. Motor cortex tSMS reduced peak functional connectivity with the stimulated and not-stimulated hemispheres (Table 1). A group by time interaction was identified in bilateral precuneus extending into paracentral lobule (Brodmann areas 7 and 31; Table 1), as well as the left superior parietal cortex. The analysis of the time course of functional connectivity strength between Brodmann area 7 and the left-M1 seed (stimulated region) showed a significant difference between real and sham groups (repeated-measures ANOVA, Time × Stimulation: F(3,30) = 6.6, p = 0.002). Specifically, left-M1 tSMS decreased the functional connectivity in these components of the SMN compared with sham in Post1 time (Bonferroni-corrected least-significant difference post-Hoc test, p = 0.009). The real group showed a significant effect of time (follow-up repeated-measures ANOVA, Time: F(3,13) = 7.051, p = 0.005), with connectivity in the Post1 being significantly lower than the connectivity in the Post3 and Post4 scanning runs (Bonferroni-corrected least-significant difference post-Hoc; Post1 vs. Post2: p = 0.135; Post1 vs. Post3: p = 0.020; Post1 vs. Post4: p = 0.003). This effect did not exist in the sham group (follow-up repeated-measures ANOVA, Time: F(3,15) = 1.508, p = 0.253) (Fig. 3B).

**Table 1:** Brain areas that showed significant changes in connectivity of the comparison between slope contrast in the real group and slope contrast in the sham group (cluster determined by z > 2.3 and a (corrected) cluster significance threshold of p = 0.050).

Cluster list						
Cluster index	Voxels	P	Z-MAX (mm)	Z-MAX X (mm)	Z-MAX Y (mm)	Z-MAX Z (mm)
2	14,810	1.67e−06	3.58	6	− 33	51
1	4132	0.0447	3.2	− 28	− 67	49

Local maxima						
Anatomical location	Cluster	Z	MNI coordinates			
			x	y	z	
Right Cerebrum.Parietal Lobe.Precuneus.Gray Matter.Brodmann area 7	2	3.58	6	− 33	51	
Right Cerebrum.Frontal Lobe.Paracentral Lobule.Gray Matter.Brodmann area 31	2	3.53	7	− 26	48	
Left Cerebrum.Parietal Lobe.Precuneus.	2	3.52	− 4	− 43	61	
Right Cerebrum.Frontal Lobe.Sub-Gyral.White Matter.	2	3.51	21	− 25	49	
Left Cerebrum.Frontal Lobe.Paracentral Lobule.White Matter.	2	3.45	− 9	− 24	54	
Right Cerebrum.Parietal Lobe.Sub-Gyral.White Matter.	2	3.41	21	− 27	48	
Left Cerebrum.Parietal Lobe.Superior Parietal Lobule.White Matter.	1	3.2	− 28	− 67	49	
Left Cerebrum.Parietal Lobe.Precuneus.White Matter.	1	3.12	− 27	− 70	41	
Left Cerebrum.Parietal Lobe.Superior Parietal Lobule.White Matter.	1	3.05	− 28	− 62	58	
Left Cerebrum.Parietal Lobe.Sub-Gyral.White Matter.	1	3.03	− 29	− 60	36	
Left Cerebrum.Parietal Lobe.Sub-Gyral.White Matter.	1	3	− 28	− 57	35	
Left Cerebrum.Parietal Lobe.Superior Parietal Lobule.Gray Matter.Brodmann area 7	1	2.96	− 12	− 58	66	

We also tested for tSMS-dependent changes in functional connectivity that habituated over time but taking PCC and left-V1 as seed regions. These two seed-based connectivity analyses (SCA) failed to show a significant group by time interaction at cluster threshold of z > 2.3.

### Resting state frequency domain power analysis

**Figure 4:**
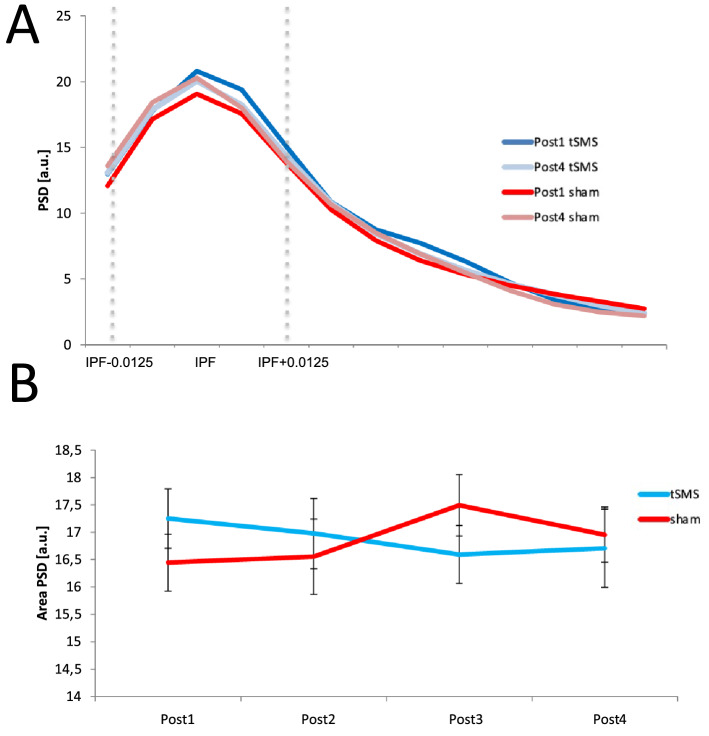
Focal resting state frequency domain power analysis. (**A**) Power spectrum density (PSD) average aligned to the individual peak frequency (IPF) calculated on the left-M1 seed of the BOLD signal extracted from resting state fMRI time series in the sham and real group during Post1 and Post4. (**B**) Time course of the area below the curve of PSD within the frequency range of the IPF ± 0.0125 Hz in the sham and real groups. Error bars depict standard error of the mean (SEM). *a.u.* arbitrary unit.

The area below the PSD curve within the frequency range of the IPF ± 0.0125 Hz was individually calculated for the left-M1 seed for the first and fourth scanning blocks for each group, Fig. 4A. The left-M1 seed showed similar PSD in real and sham groups and no effects were observed in relation to time (repeated-measures ANOVA, Time × Stimulation: F(3,30) = 0.787, p = 0.511) (Fig. 4B).

## Discussion

In the present study, we investigated the effects of 10 min tSMS on spontaneous low frequency BOLD signal fluctuations, a measure that allows characterization of RSNs. Our main result is that functional connectivity was reduced after tSMS within the stimulated network (stimulated SMN) and that power spectral characteristics of low-frequency oscillations during the resting state were not modified by tSMS in the stimulated region. As local low-frequency oscillations were unchanged (seed used for seed-based connectivity analysis), the functional connectivity modification is unlikely to be due to a simple change in local activity below the magnet^58^. Lastly, we quantified the effects induced by tSMS within networks other than the stimulated SMN. The seed-based correlation analysis (SCA) did not show any effect between real tSMS and sham in these different networks (DMN and the VN). Of course, we cannot exclude that prolonged tSMS or stronger magnetic fields may have differential effects.

SCA requires the a priori selection of a seed in a ROI. The choice of the seed will define the resulting functional connectivity network, e.g., SMN, DMN and VN. These networks are calculated with spherical seeds with a radius of 5 mm centered on coordinates based on the literature. Studies that have used different groups of subjects, non-identical seeds and different types of MR acquisition protocols, show large overlap between their results, indicating the robust formation of functionally linked resting-state networks in the brain during rest^59^. Therefore, we believe that our results do not depend on the seed we have chosen to study.

Compared to the current results, the different effects reported after tSMS over SMA (increased local resting-state activity) may be due to a specific response of each brain area^13^. On the other hand, we would like to remark that the experimental design and tSMS duration where different in their experiments with tSMS over SMA. Pineda et al.^13^ used longer stimulation period (30 min), and they stimulated simultaneously both right and left parts of the SMA.

The tSMS induced functional connectivity changes were observed only in a few clusters within the SMN, an observation with several possible interpretations. It is possible that part of the SMN is more sensitive to tSMS, but also that the sensitivity of measurement of the functional connectivity is not homogenous within the network. These results suggest that tSMS effects are influenced by—at least—a within-network interaction, which should be considered when interpreting behavioural effects of tSMS. Behavioural consequences of tSMS cannot be interpreted based only on the role of the directly stimulated regions (M1 in our experiment), and the roles of the whole functional network should also be considered. For instance, when applying tSMS over M1, the logical aim is to modulate the activity in a given motor task. However, our results suggest that using this stimulation protocol, the activity of other areas (e.g. parietal areas) is also being modulated. A recent study, using a longer time of stimulation and a stronger magnet, reported changes induced by tSMS over M1 in the EEG power spectrum at C3 and in interregional spontaneous EEG coupling between C3 and the parietal midline electrodes^60^. However, it is important to note that the results shown in the present study pertain to resting-state conditions and cannot be immediately extrapolated to other functional conditions (e.g. during a task execution). Cognitive and motor tasks are performed not by isolated brain regions but by networks, and RSNs are thought to reflect their intrinsic functional architecture^31,32^. A network consists of several functionally connected brain regions working in synergy to enable task execution. If the functional connectivity is changed by tSMS, the task execution may be different. Moreover, as variation in the strength of these networks has been demonstrated to be altered in several clinical conditions^33–35^, tSMS induced changes may also modulate pathological networks.

In previous experiments, we reported that subjects were not able to identify the real magnet and sham sessions^9,10,12,14,41^. In this experiment, there was a (non-significant) tendency to identify the real magnet and sham sessions that was not observed in these previous studies. Experimenters and subjects were blind to the kind of stimulation performed. Thus, we cannot exclude that the tendency to identify the real tSMS is due to a summation of the effects of the real tSMS and the magnetic field (plus radiofrequencies) of the MRI (see also below). Future studies will try to clarify this aspect.

The present study has a number of limitations. Our functional connectivity analyses were done with average measures across about 5 min periods after the tSMS; therefore, dynamic fluctuations in the functional connectivity during the tSMS are not evaluated. Another factor to take into account when examining tSMS effects we reported here is that we cannot exclude a summation of the effects of the real tSMS (focal) and the homogeneous magnetic field (plus radiofrequencies) of the MRI. We evaluated only the effects of one magnetic field intensity and one duration (10 min), so we cannot exclude that prolonged tSMS or stronger magnetic fields may have differential effects.

## Conclusions

In the present study we evaluated the after-effects of tSMS on functional connectivity and on spontaneous low frequency signal fluctuations by applying tSMS over the left M1 of healthy subjects. We found reduced connectivity within the stimulated network. These network interactions should be taken into account when using tSMS for studying brain function and behaviour in health and disease.

## Data Availability

The datasets used and analyzed during the current study are available on request from the corresponding author.
